# Amoebic Liver Abscess and Indigenous Alcoholic Beverages in the Tropics

**DOI:** 10.1155/2018/6901751

**Published:** 2018-07-10

**Authors:** T. Kumanan, V. Sujanitha, S. Balakumar, N. Sreeharan

**Affiliations:** ^1^Department of Medicine, Faculty of Medicine, University of Jaffna, Jaffna, Sri Lanka; ^2^Department of Biochemistry, Faculty of Medicine, University of Jaffna, Jaffna, Sri Lanka

## Abstract

Amoebic liver abscess (ALA) seen commonly in the tropics is predominantly confined to adult males, especially those who consume locally brewed alcohol, although intestinal amoebiasis occurs in all age groups and in both genders. Whether the role of alcohol in the development of ALA is incidental and casual or whether alcohol is causally implicated has been debated. It has been argued that socioeconomic factors and poor sanitary conditions are the primary culprits that casually link alcohol to ALA. However, there has emerged an abundance of data that implicates alcohol in a more causal role in facilitating the extraintestinal invasion of the infective protozoan and the subsequent development of ALA. These factors include the role of alcohol in host immunity, parasitic proliferation, and invasion and in creating a conducive hepatic microenvironment. The contributory role of alcohol-induced increase in hepatic iron stores and lipid content is discussed. Late-stage liver disease with fibrosis seems to be protective for the development of ALA. Further research is necessary to elucidate the many possible mechanisms that predispose to hepatic amoebiasis, so that appropriate individual and population-based preventive measures can be implemented.

## 1. Introduction

Liver abscess may result from a variety of pathogenic organisms including bacteria, fungus, protozoa, and parasites. It is a common clinical entity encountered by physicians throughout the world. One of the early reports states that two-thirds of its aetiology in the tropics could be attributed to amoebic liver abscess (ALA); in contrast, three-fourths in the developed countries are attributed to bacterial pathogens [1].

ALA is very common in many Asian countries including Sri Lanka. Incidence of ALA in other continents is mainly due to* Entamoeba histolytica* infection acquired during travelling. The prevalence of ALA in different places across the world is shown in Table 1.

ALA caused by* Entamoeba histolytica* is presently the third most common cause of death from parasitic diseases [2]. Susceptibility to this infection and the clinical outcomes are variable in the vulnerable population. Most of the studies from tropics illustrate the fact that ALA is extremely common among males who consume alcohol beverages, in particular the indigenous variety. This interesting and important association raises a query: Is this relationship casual or causal?

## 2. Amoebic Liver Abscess and Alcohol

A close relationship between the consumption of indigenously brewed alcohol beverages and the occurrence of ALA in the tropics has been established over a period of time by several large-population studies [3, 4, 5]

Reasons for this association between local alcohol beverages and ALA could be multifactorial. Factors influencing the association could be related to the pathogen, contents of beverages, status of the liver, and the immunity of the host. They are more vulnerable perhaps due to the large infective dose of* Entamoeba histolytica* and other bacterial pathogens ingested with the unhygienically brewed beverage. Associated nutritional status of the population and poor sanitation could also play a pivotal role. Hai et al. stated that alcohol-induced hepatic dysfunction and possible suppression of amoebistatic immune mechanisms by substances in the beverages could also be attributed in the mechanism [6].

Mukhopadhyay et al. [5] have suggested that “alcohol can predispose to ALA through a multitude of mechanisms, including hepatic damage by alcohol, lowered body resistance and suppression of liver function due to poor nutritional status of habitual consumers of alcohol, increased presence of amoebae in the liquor prepared locally with poor regard to aseptic procedures, and depression of immune mechanisms in chronic alcoholics.” A study conducted in India in 2011 [4] showed that 67.5% of patients with amoebic liver abscess are from the low socioeconomic class and 72% were alcoholics. It was also noted that alcoholics had larger abscesses, a greater frequency of complications, and delayed resolution of the abscesses.

### 2.1. Age and Gender Differences in Amoebic Liver Abscess

Many studies have confirmed the fact that ALA is extremely common in adult males. Naturally the question whether this could be explained by the gross cultural disparity observed in drinking habits between adult males, females, and children or whether other possible aetiological mechanisms exist to explain the gender difference arises.

Amoebic liver abscess is extremely rare in children and infection is mainly restricted to the gut [7]. Malnutrition predisposes children to infection and the nutritional hormone leptin is important in the immunity to the enteric pathogen* Entamoeba histolytica* [8]. Malnourished children are known to have low levels of the hormone leptin that signals satiety and also influences the immune system. Genetic variants in leptin and the leptin receptor were evaluated for association with infection, and increased susceptibility to intestinal* Entamoeba histolytica* infection was found to be associated with a single amino acid polymorphism in the leptin receptor in the children studied in a Bangladeshi cohort of amoebiasis [9].

Although intestinal amoebiasis and asymptomatic amoebic infections are common among children and females, why ALA is extremely uncommon in these population is unanswered and a matter of debate.

The incidence of amoebic liver abscess in males increases after puberty and the peak incidence is approximately at the age of 40 years [7]. A study was done to examine the gender differences in immunocompetent mice to amoebic infection of the liver and found that 75% of male mice developed abscess, while none of the female mice did so. It also revealed that female mice cleared the infection faster and there were clear differences in the production of cytokines in both categories, which could explain the differences in the infection rate in both genders [7]. No human studies have been done to elicit this gender difference to date.

Several studies have highlighted the strong association of alcohol intake, mainly of the indigenous variety, in the preponderance of males developing ALA. A study in Bangladesh found that the male-to-female ratio was 21:1 and a habit of indigenous alcohol consumption was observed in four-fifths of the patients with ALA [10]. A Sri Lankan study recently found that almost all the 346 confirmed ALA patients were males and consumed alcohol beverages, in particular the indigenous variety [11]. Similar observation was made in an Indian study [6] conducted in the early 80s among 220 patients with ALA. The majority of the subjects were young or middle-aged males and 85% gave a history of drinking toddy, a local palm wine of the region.

However, Reddy and Thangavelu [12] proposed alternative mechanisms to explain the gender difference, suggesting that “the female menstrual cycle prevents hepatic congestion and thus makes the organ less susceptible to abscess formation. Additionally, a relative iron deficiency due to menstruation and protective hormonal factors in women of child bearing age limits* Entamoeba histolytica* invasion in females.” A laboratory-based study [13] found that testosterone increased the susceptibility of mice to* Entamoeba histolytica* liver abscess by reducing the interferon-gamma secretion by natural killer cells. Therefore, these alternative, non-alcohol-based mechanisms could also explain the relative preponderance of ALA in males.

### 2.2. Amoebic Liver Abscess and Alcohol: Is It a Casual Association?

We will initially explore the factors that point towards a casual relationship with alcohol and other factors, primarily related to socioeconomic status and personal hygiene possibly being the primary culprits. As most of the studies clearly point out, ALA almost always occurs in males with a history of drinking indigenously brewed alcohol beverages in a poor socioeconomic background. As the primary contributory factor is the male gender, we need to explore whether this gender preponderance is purely due to alcohol-related factors or whether it is due to other factors including the level of immunity of the host.

How amoeba gets into the beverage is not well understood. Most likely this is due to the unhygienic practices commonly seen at the drinking taverns or cottages in many tropical countries. The important finding observed in most of the studies is that majority of ALA patients are from a poor socioeconomic background and are manual laborers [10, 6, 11]. Poor hygiene has been associated with increased risk of amoebic liver abscesses and is directly proportionate to disease progression and extent of liver injury [14]. Parasite cysts are transmitted through contaminated food and water, making the incidence of disease high in areas of poor sanitation. Good hygienic practices cannot be expected at these places as the majority of subjects are male farmers from lower- and middle-class backgrounds in one study [15] and 68% belonged to low socioeconomic group in another study [11]. The prevalence of asymptomatic intestinal amoebiasis is also common in these regions, although the prevalence rate varies with the methods used for screening. It is high when microscopic examination method is employed and low with the use of new molecular techniques [16]. It can be assumed that the regular visitors to these drinking places could be the source of pathogen by unhygienic practices including open air defecation, poor hand washing practices, unwashed utensils at these taverns, and the consumption of unboiled drinking water. Indeed, some studies have documented the prevalence of vectors of amoebiasis, including flies and cockroaches at these places [17]. The study done in northern Sri Lanka [11] showed heavy alcohol consumption, mainly toddy, in all 346 confirmed patients with amoebic liver abscesses but none of the cultured toddy samples grow* Entamoeba histolytica.*

All the above considerations may suggest that the relationship between alcoholic beverages and ALA is incidental and casual.

### 2.3. Alcohol as a Causal Factor of Amoebic Liver Abscess

We now turn to the accumulation of evidences which suggests that the relationship between alcohol consumption and ALA is not incidental and casual but causal in nature. The beverage and its constituent ethanol could influence several factors to make individuals infected with amoeba to develop extraintestinal invasion. These factors could be pathogen- or host-related.

### 2.4. Alcohol and Immunity

Immunological mechanisms of the host are important in combating a protozoal pathogen. The extensive nature of amoebic liver abscess in patients with HIV infection proves that cell-mediated immunity is very important in controlling the disease [18]. Furthermore, animal studies have shown that immune suppression after thymectomy or splenectomy resulted in an increased incidence of amoebic liver abscess [19].

Alcohol has a significant influence on both cell-mediated and humoral immunity. Ethanol abuse is an important cause for reactivation of tuberculosis in the tropics [20]. There is ample evidence to suggest that both acute alcohol consumption and chronic alcohol consumption suppress almost all the components of the immune system, including initial response to a pathogen and the tumor surveillance system [21–23]. Alcohol suppresses function of Kupffer cells in liver, which has important role in clearing the amoebae.

### 2.5. Alcohol and* Entamoeba *Proliferation

*Entamoeba* can be cultured in both xenic and axenic culture media. Boeck and Drbohlav first introduced xenic culture of* Entamoeba* in 1925 in a diphasic egg slant medium, and a modification of this medium (Locke-egg) is still used today [24]. A culture of parasites grown in association with an unknown microbiota is called xenic culture. Whether palmyrah wine/toddy provided a similar medium for the proliferation of* Entamoeba histolytica* is yet to be established. It is reasonable to infer such a possibility as the constituents of palm wine provide ample nutrients in the form of disaccharides and enormous amount of yeast cells to feed on. The acetic acid could provide an optimum mild acidic pH for its optimum growth. Mortan et al. have found that the components of jiggery made up of palmyrah palm of northern Sri Lanka include “1.04% protein, 0.19% fat, 76.86% sucrose, 1.66% glucose, 3.15% total minerals, 0.861 % calcium, and 0.052% phosphorus and also 11.01 mg iron per 100 g and 0.767 mg of copper per 100 g” [25, 26]. The alcohol content of toddy is 4.5-7%. As palmyrah toddy contains both iron and alcohol, both these constituents can have a synergistic action to cause ALA. Therefore, palmyrah toddy provides a more favourable microenvironment than distilled alcoholic beverage, which has no iron in its composition, for the proliferation of amoeba, thereby facilitating the pathogenesis of ALA.

Alcohol-induced hyperpermeability and dysbiosis of intestinal bacteria may both contribute to the pathological effects of alcohol, permitting the entry of* Entamoeba histolytica* into the blood [27].

A study found that exacerbated inflammatory milieu is established and contributes to liver tissue damage and probably supports the survival of parasites. IL-1*β* was detected mainly in neutrophils and macrophages from the periods of acute infection to subacute and chronic infection and decreased when granuloma was formed [28].

Parasite genotype is also a contributory factor for the causation of amoebic liver abscess. Studies have confirmed that intestine and liver abscess amoebae are genetically distinct. This suggests that either* Entamoeba histolytica* subpopulation in the same infection shows varying organ tropism or DNA reorganization event takes place prior to or during metastasis from intestine to liver [29].

Several host-related factors could contribute to its invasiveness in the liver including the architecture, fat content, and hormones. Several other factors contribute to the pathogenicity of* Entamoeba histolytica* and some may still await identification. Three important pathogenic factors that have been investigated and characterized are at molecular level: (1) the Gal/GalNAc adhesion, mediating adherence to host cells and contributing to amoebic resistance to complement, (2) the amoebapores, small peptides that produce pores in target membrane, and (3) cysteine proteinases that play a key role in* Entamoeba histolytica* tissue invasion, evasion of host defence, and parasite induction of inflammation [30]. Beyond doubt* Entamoeba histolytica* genome sequence and new molecular techniques will rapidly advance the understanding of the epidemiology and pathogenicity of amoebiasis. And also further work needs to be done to see whether creating a microbiological and biochemical environment similar to palm toddy enhances the growth of* Entamoeba histolytica* in culture media.

### 2.6. Alcohol and Liver

Although alcohol is consumed by many, serious alcoholic liver disease occurs in susceptible people only. The deleterious effects of alcohol on liver are influenced by heredity, gender, diet, and other coexisting liver diseases. Liver injury by alcohol is mainly due to its metabolites due to direct toxicity or by inflammation induced by them. These types of continuous liver injury can lead to fibrosis and cirrhosis later [31].

Several factors related to the status of liver could also contribute a favourable environment to the amoeba when it reaches the liver. It is interesting that only small numbers of amoeba have been identified relative to the size of the abscess, which suggests that* Entamoeba histolytica* can kill hepatocytes effectively with extensive tissue destruction [32]. Studies have pointed out that chronic stage of ALA is characterized by defective cell-mediated immunity and the suppression of T cells and their defective proliferative responses [33]. Experimental studies have revealed the fact that presence of iron promotes the growth of amoeba in vitro. [26] Chronic ethanol consumption has been well documented to increase the hepatic iron stores [34]. It is therefore fair to postulate that high hepatic iron content is an important contributory factor to develop ALA, although the exact mechanisms are yet to be established [35]. Possible mechanisms include hepcidin, a key hormone in iron metabolism which inhibits intestinal iron absorption and the release of iron from macrophages. Alcohol has been shown to downregulate hepcidin expression in the liver, leading to elevated expression of iron transporter proteins in the duodenum [34]. Furthermore, another study [36] demonstrated that iron chelation interrupts the completion of the fermentation pathway of* Entamoeba histolytica* by removing the metal cofactor indispensable for the functional stability of alcohol dehydrogenase, thus affecting trophozoite survival. Hepatic tissue injury is directly correlated to the duration and amount of iron loading. Iron is an important factor for* Entamoeba histolytica* growth and virulence and also an important factor in the adherence and cytotoxicity of* Entamoeba histolytica*, acting by modulation of gene expression of virulence factors [37, 38]. Thus, the above iron-based mechanisms together with increased iron content of palmyrah toddy [25] as opposed to nontraditional alcoholic spirits would favour a specific causal role for the indigenous beverage to enhance hepatic iron content and provide a conducive environment for* Entamoeba histolytica* growth.

Another study found that the incidence of liver abscess in the cirrhotic individuals was low, despite their increased susceptibility to infections due to diminished immunity [39]. The postulated mechanisms are many.* Entamoeba histolytica* lacks mitochondria and hence survives by scavenging nutrients from the host cells. The organism is rich in phospholipases and lysophospholipase essential for cytolysis of cells [40]. Hypocholesterolemia has been recorded in patients with ALA [41]. Fatty Liver is high in intracellular triglycerides and is an organ active in the production of cholesterol. Cholesterol increases the virulence of* Entamoeba histolytica* in animal models and in vitro culture. It is also likely that as the fatty liver disease progresses to cirrhosis, the environment to favour residence and multiplication of* Entamoeba histolytica* is lost due to both loss of nutrients from the hepatocytes and the “loss of space” as a result of fibrotic scarring, hence the rarity of cirrhosis and ALA.

## 3. Conclusion

This review has highlighted the strong association between the consumption of alcohol, particularly of the indigenous variety such as palmyrah toddy, and the development of hepatic amoebiasis. Although the link with alcohol could be casual with other socioeconomic and personal hygienic factors contributing to this association, there appears to be overwhelming evidence to suggest a more causal role for alcohol. It is likely that alcohol creates an immune and biochemical microenvironment that enhances extraintestinal invasion and settlement in the liver of* Entamoeba histolytica* and transforms asymptomatic and symptomatic intestinal amoebiasis into hepatic amoebiasis, a disease that is common in the tropics and a major cause of morbidity and mortality. Since humans are the only significant reservoir* of Entamoeba histolytica *infection, it is essential to identify the modifiable causes especially in developing countries so that the disease burden can be reduced or even eliminated. Further research is necessary to elucidate the many possible mechanisms that predispose to hepatic amoebiasis so that appropriate individual and population-based preventive measures can be implemented.

## Figures and Tables

**Table 1:** Prevalence of ALA in different places across the world.

**Region/country**	**Origin**	**Period**	**Total number of ALA cases**	**References**
**Bangladesh**	Hospitals in northern district	2008–2010	90	Alam et al. 2014 [10]
**Sri Lanka**	Teaching Hospital-Jaffna	2012–2015	367	Kannathasan et al. 2018 [3]
**Bordeaux, France**	Bordeaux University Hospital Centre	1995–1999	20 (18 HIV+)	Djossou et al. 2003 [42]
**Thailand**	Nationwide hospital admission data	2008–2013	448	Poovorawan et al. 2016 [43]
**Toronto, Canada**	Seven hospitals in Toronto	1980–2005	29	Wuerz et al. 2012 [44]
**Sonora, Mexico**	4 hospitals in Hermosillo	2000–2005	319	Valenzuela et al. 2007 [45]
**San Francisco, USA**	Medical histories	1979–1994	56	Seeto and Rockey 1999 [46]
